# 2-Pentanone Production from Hexanoic Acid by *Penicillium roqueforti* from Blue Cheese: Is This the Pathway Used in Humans?

**DOI:** 10.1155/2014/215783

**Published:** 2014-03-27

**Authors:** Valerie Walker, Graham A. Mills

**Affiliations:** ^1^Department of Clinical Biochemistry, Southampton General Hospital, C level, MP 6, South Block, Tremona Road, Southampton SO16 6YD, UK; ^2^School of Pharmacy and Biomedical Sciences, University of Portsmouth, St Michael's Building, White Swan Road, Portsmouth PO1 2DT, UK

## Abstract

Production of 2-pentanone, a methylketone, is increased in fasting ketotic humans. Its origin is unknown. We hypothesised that it is formed via **β**-oxidation of hexanoic acid by the peroxisomal pathway proposed for methylketone-producing fungi and yeasts. We used *Penicillium roqueforti* cultured on fat (margarine) to investigate 2-pentanone production. Headspace gas of incubates of the mould with a range of substrates was analysed using solid-phase microextraction with gas chromatography-mass spectrometry. Consistent with the proposed pathway, 2-pentanone was formed from hexanoic acid, hexanoyl-CoA, hexanoylcarnitine, and ethyl-3-oxohexanoic acid but not from ethylhexanoic, 2-ethylhexanoic, octanoic, or myristic acids, octanoylcarnitine, or pentane. However, the products from deuterated (D) hexanoic-D_11_ acid and hexanoic-2, 2-D_2_ acid were 9D- and 2D-2-pentanone, respectively, and not 8D- and 1D-2-pentanone as predicted. When incubated under ^18^O_2_/^14^N_2_, there was only a very small enrichment of [^16^O_2_]- with [^18^O_2_]-containing 2-pentanone. These are new observations. They could be explained if hydrogen ions removed from hexanoyl-CoA by acyl-CoA oxidase at the commencement of **β**-oxidation were cycled through hydrogen peroxide and reentered the pathway through hydration of hexenoyl-CoA. This would protect other proteins from oxidative damage. Formation of 2-pentanone through a **β**-oxidation cycle similar to *Penicillium roqueforti* would be consistent with observations in humans.

## 1. Introduction 

When investigating patients for suspected inherited metabolic disorders, we selectively include a qualitative analysis of volatile compounds in the urine screen, generally when patients have presented with ketosis, encephalopathy, hypoglycaemia, or an abnormal body odour. We commonly find small amounts of the methylketone, 2-pentanone. In a review of the profiles of volatile compounds of approximately 400 patients investigated from 1999 to 2008, we identified 23 patients whose excretion was clearly increased at presentation. Sixteen patients had ketonuria. Fourteen patients had recurrent vomiting or fasting hypoglycaemia and four had decompensated inherited disorders of fatty acid *β*-oxidation. The abnormality had resolved in follow-up samples and hence was transient. In one adult, urinary 2-pentanone decreased to 15% of the fasting value two hours after a glucose load in association with a fall in the plasma ketone 3-hydroxybutyrate to 9% of the fasting value [1]. Others have identified 2-pentanone in urine from normal adults [2, 3] and reported increased excretion by healthy ketotic adults after prolonged fasting [4] and by rats after fasting [5] or with alloxan-induced diabetes [6]. The source of 2-pentanone in humans remains unknown but has been assumed to be from increased fatty acid *β*-oxidation [3, 5].

**Figure 1:**
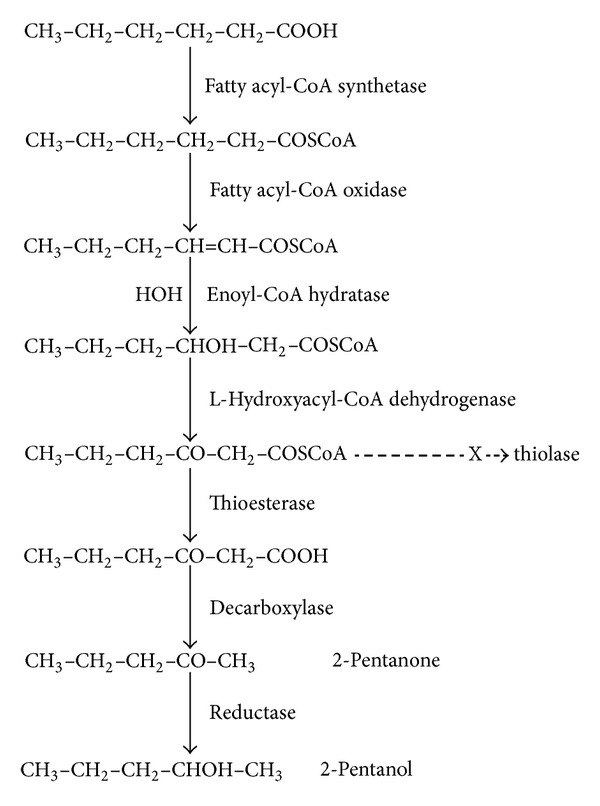
Widely accepted pathway for the production of 2-pentanone by* Penicillium roqueforti. *

By chance, we noticed that a heavy growth of fungus was causing lipolysis of margarine which had been contaminated accidentally with blue cheese. The fungus was also producing large amounts of methylketones which included 2-pentanone. Cultures from the contaminated margarine and cheese grew the same organism, which was* Penicillium roqueforti. *Many filamentous fungi, including members of* Penicillium *sp. and* Aspergillus *sp., produce methylketones when grown on fatty acids as the sole energy source [7]. Production by* Penicillium roqueforti* has been exploited by the cheese industry to give blue cheese its characteristic flavour. Medium-chain fatty acids are shortened by one carbon unit during conversion to a series of methylketones generally with abundance of 2-heptanone > 2-nonanone > 2-pentanone > 2-undecanone, although there is variation between cultures [8–10]. Despite its long-standing commercial application, the biochemical pathway involved has not been demonstrated unequivocally. From the collective evidence, methylketones appear to be formed by a futile cycle associated with incomplete *β*-oxidation of fatty acids. It is proposed that *β*-oxidation proceeds normally to the formation of medium-chain 3-oxoacyl-CoA intermediates but is then halted at the medium-chain acyl-CoA thiolase reaction. Thioesterase(s) release Coenzyme A (CoASH) from the accumulating intermediates and the 3-oxoacids are decarboxylated to methylketones. CoASH might then be recycled to initiate oxidation of more fatty acids, but their metabolism would again be held up at the thiolase bottleneck [7, 8, 11, 12]. The process probably occurs by the peroxisomal *β*-oxidation pathway and not by mitochondrial *β*-oxidation.* Penicillium roqueforti *has peroxisomes (microbodies) and methylketone production by other fungi is associated with large increases in the number of peroxisomes characterised by catalase [13].  Figure 1 shows the proposed pathway for production of 2-pentanone from hexanoic acid.

It seemed feasible that 2-pentanone was produced in ketotic humans by a similar pathway to that used in filamentous fungi. The aim of this study was to investigate 2-pentanone production from fatty acids further, using* Penicillium roqueforti *cultured on margarine as a readily accessible source of a methylketone-producing organism. Preliminary observations supported the scheme outlined in Figure 1, but experiments with deuterated hexanoic acid did not yield the anticipated deuterated metabolites. This new observation is the focus of this report. Although apparently contradictory to the *β*-oxidation hypothesis, we suggest that the findings are consistent with the recently elucidated mechanism of hydration of enoyl-CoA fatty acids in the *β*-oxidation pathway [14, 15] and hence support the case.

## 2. Materials and Methods 

### 2.1. Reagents and Consumable

Gas mix 1 : 3 ^18^O_2_ oxygen (98%) and ^14^N_2_ nitrogen was from CK Gas Products Ltd. (Hook, UK). Hexanoic acid-2, 2-D_2_ acid (98 atom % D); hexanoic-D_11_ acid (98 atom % D); hexanoyl-CoA trilithium salt hydrate (C6:0); hexanoylcarnitine, hexanoic acid, octanoic acid, 4-methyl-2-pentanone (MIBK) >99%, cyclohexanone 99.8%, 2-pentanone 99.5%, 2-pentanol 98%, ethyl 3-oxohexanoate (ethyl butyrate) 98% purities, and pentane were from Sigma-Aldrich (Gillingham, UK). Hexane (HPLC grade) was from Fisher Scientific UK Ltd. (Loughborough, UK). Blue cheese and margarine were obtained commercially. In preliminary studies (Section 3.2), 250 *μ*L of a 1 in 2,000 dilution of cyclohexanone in water was added as internal standard (IS). In later experiments (Sections 3.3 and 3.4), the IS was 20 *μ*L of a 1 in 1,000 dilution of MIBK in water. Working solutions of hexanoic acid (^1^H or deuterated) were prepared as dilutions in melted margarine (1 *μ*L/mL; 8 mmol/L).

Carboxen-polydimethylsiloxane 75 *μ*m film thickness solid-phase microextraction (SPME) fibres and SPME fibre syringe holders were from Supelco (Poole, UK); headspace (HS) vials (22 mL) with soft silicone rubber seals (20 mm diameter) and aluminium caps were from Alltech Associates Ltd. (Carnforth, UK). Water was deionised by reverse osmosis.

### 2.2. Gas Chromatography-Mass Spectrometry

The bench-top gas chromatograph-mass spectrometer (GC-MS) system was a 6890N GC linked to a 5973 quadrupole MS (Agilent Technologies, Bracknell, UK) fitted with a Rt-BetaDEXse chiral fused-silica capillary column (30 m × 0.25 mm I.D., film thickness 0.25 *μ*m; Thames Restek, Saunderton, UK) and a narrow bore (0.75 mm) SPME injector liner (Supelco). The GC-MS system operating conditions were as follows: carrier gas helium, flow rate 0.6 mL/min; splitless injection; no solvent delay; injector temperature 250°C; interface transfer line temperature 280°C; oven temperature programme 40°C (5 min) then 5°C/min to 210°C. The MS system was operated in electron ionisation (70 eV) mode and scanned from 10 to 400 amu. Compounds were identified by reference to authentic standards and/or the NIST05a mass spectral library.

### 2.3. Samples and Sample Preparation

Small (2-3 mm) fragments of blue cheese were placed onto the surface of a thick layer of margarine, covered, and left in the dark at room temperature. Growth was evident (by naked eye) by around 4 days and was florid from 8 days. Between 8 and 21 days, the surface layer of fungus with a small amount of attached margarine was transferred to a sterile beaker and mixed vigorously using a rotary mixer. Aliquots of approximately 1.5 g were transferred to HS vials and weighed accurately. In most experiments, 100 *μ*L of working hexanoic acid (800 nmol/vial) and 400 *μ*L of melted margarine were added to the test vials and 500 *μ*L of melted margarine to control vials. Additions of other compounds were as described in Section 3. IS was added for semiquantitative analyses. After adding a magnetic stirrer bar, the vials were capped and crimp-sealed with a silicone rubber septum. Calibrants of 93 nmol/vial of 2-pentanone with IS in 1.5 g of margarine were used to quantify the traces of 2-pentanone present basally in margarine without any additions and in time course and linearity experiments.

### 2.4. Headspace-SPME Procedure

The HS gas was analysed using the validated procedure of Mills and Walker [16], with minor modifications. Vials were mounted on a magnetic stirrer (Variomag compact magnetic stirrer, model 40151, CamLab, Cambridge, UK) submerged in a water bath at 50°C (Grant stirred thermostatic bath, CamLab) and stirred at approximately 500 rpm. Volatiles were extracted for 30 min onto a Carboxen-polydimethylsiloxane SPME fibre inserted through the vial septum. The extracted compounds were then desorbed from the fibre (3 min) in the injector port of the GC-MS. To avoid carry-over, the SPME fibres were conditioned between runs for 6 min at 300°C in the injector port of a separate GC. For semiquantification, the ratios of peak areas of metabolites to IS per vial were calculated.

## 3. Results and Discussion 

### 3.1. *Penicillium roqueforti* Cultures

Cultures of blue cheese on margarine always yielded a good growth of methylketone-producing fungus. In some preparations, there were visible small zones of margarine liquefaction indicating lipolysis. Application of 3% hydrogen peroxide (H_2_O_2_) caused vigorous bubbling of oxygen indicating catalase production. Margarine has not been reported as a culture medium for* Penicillium roqueforti, *but incubation with butter or milk fat together with lipase has been used to accelerate flavour development in blue cheese [8, 9]. Release of free fatty acids by lipase activity is rate limiting to synthesis of methylketones and correlates with their production [8, 9].

### 3.2. 2-Pentanone Production from Hexanoic Acid and Related Metabolites

Preliminary experiments were undertaken to identify compounds which are converted to 2-pentanone. Despite vigorous mixing prior to transfer to the HS vials, the fungal samples were not homogeneous and accurate quantification was impossible. Increases were assessed approximately by comparing the ratios of the peak areas of 2-pentanone relative to IS in vials with added compounds, to the ratios in vials with added margarine only (controls). From time course experiments with hexanoyl-CoA (*n* = 2), 2-pentanone production increased progressively to 24 h. Most samples were analysed after incubation for 8 to 24 h depending on our work schedule.

#### 3.2.1. Controls

Small amounts of methylketones from hydrolysed *β*-ketoglycerides are present in heat treated milk [17]. However, if present in the margarine, these made a negligible contribution to methylketone production in our experiments; there were only traces of 2-pentanone (mean 1.0 nmol/vial, range 0.5–1.4, *n* = 10) in margarine incubated without any additions and analysed with the routine procedure, and 2-pentanol and other methylketones were not detectable. In 26 incubates with cultured blue cheese, but no additives except IS, methylketones always dominated the chromatograms of the HS volatiles. The largest peaks were 2-heptanone and 2-nonanone, generally followed by 2-pentanone and a small peak of 2-undecanone and, in some incubates, by a trace of 2-tridecanone. However, the amount of 2-pentanone varied considerably between batches of mould incubates. Some had only traces of 2-pentanone, despite having large peaks of other methylketones. In 10 experiments which included calibrants, estimates of 2-pentanone ranged from approximately 15 to 560 (median 100) nmol/vial. The concentrations of 2-pentanol were always lower. In 18 of the 26 control incubates, the median ratio of 2-pentanol to 2-pentanone was 8% (observed range 1–57%), 2-pentanol was undetectable in two incubates, and in six there was interference from 3-hydroxy-2-butanone.

#### 3.2.2. Incubates of* Penicillium roqueforti* with Compounds Which Increased 2-Pentanone Production

A series of experiments demonstrated that incubation with the following compounds increased 2-pentanone production.

*Hexanoic Acid. *With 800 nmol/vial there was a 1.2- to 3.3-fold increase in 2-pentanone (*n* = 7); the increase was linear to 1,600 nmol/vial (*n* = 2).

*Hexanoyl-CoA. *2-Pentanone and 2-pentanol increased progressively with hexanoyl-CoA additions to 2,820 nmol, with conversion to 2-pentanone plus 2-pentanol ranging from 9 to 16% (*n* = 1). With 1,410 nmol of hexanoyl-CoA/vial, there were 4.5-, 6.2-, and 31.6-fold increases in 2-pentanone (*n* = 3).

*Ethyl 3-Oxohexanoic Acid (EOH). *EOH is decarboxylated to 2-pentanone. Some decarboxylation occurs nonenzymically during analysis. Production by the fungus was calculated after subtracting 2-pentanone produced by EOH in 1.5 g margarine to correct for analytical decarboxylation. There were 1.6- and 9.7-fold increases in 2-pentanone with 630 nmol and 1,260 nmol of EOH/vial, respectively (*n* = 2).

*Hexanoylcarnitine.* With 6,300 nmol/vial, there were 3.0- and 4.6-fold increases in 2-pentanone (*n* = 2).

#### 3.2.3. Incubates of* Penicillium roqueforti *with Compounds Which Did Not Increase 2-Pentanone Production

In other experiments, incubation with the following compounds did not increase 2-pentanone production.

*Ethylhexanoic acid* (605 nmol/vial; *n* = 1), 2-*ethyl hexanoic acid* (630 nmol/vial; *n* = 1),* octanoic acid* (1,262 nmol/vial; *n* = 1),* octanoylcarnitine *(5,800 nmol/vial; *n* = 1),* myristic acid* (440 (*n* = 1) and 1,095 (*n* = 2) nmol/vial), and pentane (8,680 nmol/vial; *n* = 2). There were 2.7- and 6.8-fold increases in 2-heptanone in the octanoic and octanoylcarnitine incubates, respectively. No increases in C_7_ to C_13_ methylketones were observed with myristic acid incubation. Collectively, these observations were consistent with the concept that methylketone production by* Penicillium roqueforti *proceeds through fatty acid catabolism via the abortive *β*-oxidation pathway shown in Figure 1. They are not consistent with methylketone production via fatty acid biosynthesis, which has been proposed recently for their generation by the wild tomato species* Solanum habrochaites* [18] or by direct oxidation of pentane [19]. We observed lipolysis during culture, indicating lipase production. We found that hexanoic acid and hexanoyl-CoA both increased 2-pentanone. Production from hexanoylcarnitine probably occurred as a result of hydrolysis to hexanoic acid, since this ester is not an intermediate in the *β*-oxidation pathway. Decarboxylation of EOH to 2-pentanone was evidence of *β*-ketoacyl decarboxylase [20, 21] required for the final step of the proposed pathway. Like others, we found that methylketone production is apparently limited to one cycle of the *β*-oxidation pathway [11, 12], since octanoic acid was only chain-shortened by one carbon unit to 2-heptanone and did not generate 2-pentanone. Like us, others have reported that myristic acid was not converted to methylketones [22].

### 3.3. 2-Pentanone Production from Deuterated Hexanoic Acid

**Figure 2:**
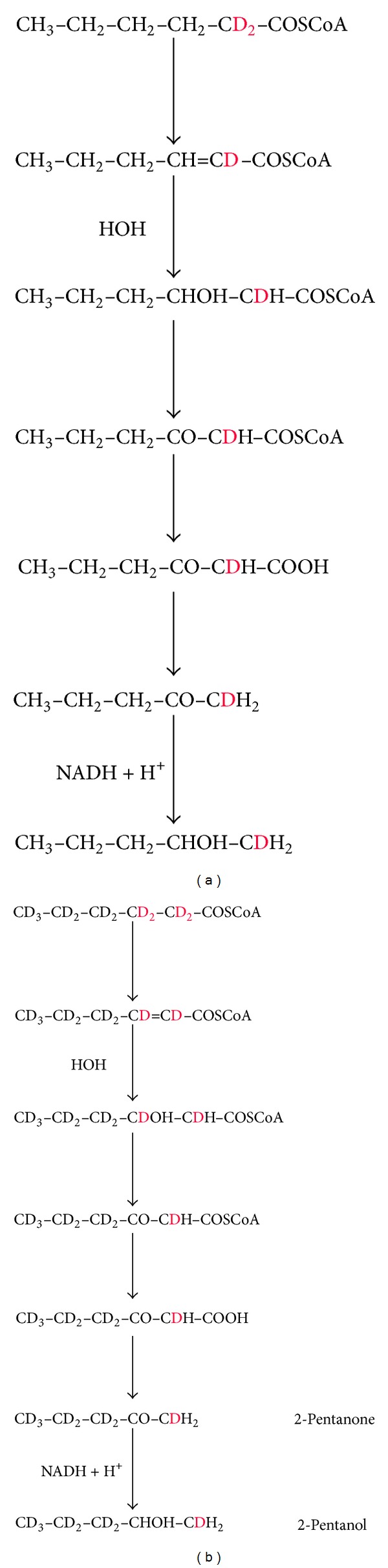
Predicted deuterated products from *β*-oxidation of (a) hexanoic-2, 2-D_2_ acid; (b) hexanoic-D_11_ acid.

Although providing support for the pathway, these observations did not add to published data. To look for new evidence, we investigated 2-pentanone and 2-pentanol production by* Penicillium roqueforti *during incubation with two preparations of hexanoic acid in which hydrogen on C2 and C3 was replaced by deuterium. To our knowledge, this has not been reported before. The predicted products from hexanoic-D_11_ acid would have eight deuterium atoms and those from hexanoic-2, 2-D_2_ acid would have one. In all the products, the C1 methyl group would be CDH_2_ (Figure 2).

**Figure 3:**
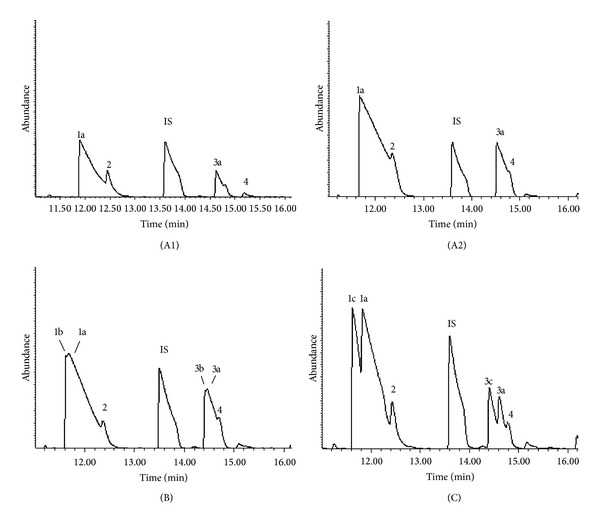
Total ion chromatograms of headspace gas from* Penicillium roqueforti* (in blue cheese) grown on margarine for 10 days and then incubated with or without added hexanoic acid in gas-tight vials for 22.8 h at room temperature. Figure (A1): no hexanoic acid added; Figure (A2): 100 *μ*L of ^1^H-hexanoic acid added; Figure (B): 100 *μ*L of hexanoic-2, 2-D_2_ acid added; Figure (C): 100 *μ*L of hexanoic-D_11_ acid added. Peaks: 1a: ^1^H 2-pentanone; 1b and 1c: deuterated 2-pentanone; 2: unidentifiable peak; 3a: ^1^H 2-pentanol; 3b and 3c: deuterated 2-pentanol; 4: 3-hydroxy-2-butanone; IS (internal standard) = 4-methyl-2-pentanone (MIBK).

In separate experiments, aliquots of blue cheese cultures on margarine were incubated with 800 nmol/vial of hexanoic-D_11_ acid on 32 occasions and with 400 nmol on three and in eight experiments with 800 nmol of hexanoic-2, 2-D_2_ acid. Incubations without additions, or with 800 nmol of ^1^H-hexanoic acid, were analysed as controls. Incubation times ranged from 6 to 22.8 h.  Figure 3 shows the total ion chromatograms for samples analysed in one experiment. These are representative of all the findings. Both 2-pentanone and 2-pentanol increased with added hexanoic acid. 2-Pentanone was always the major metabolite. Both forms of deuterated hexanoic acid produced deuterated 2-pentanone and 2-pentanol. Although these coeluted with the unlabelled metabolites produced simultaneously by the fungus from fatty acids in the margarine, separate peaks were evident and were clearly identifiable for hexanoic-D_11_ acid.

**Figure 4:**
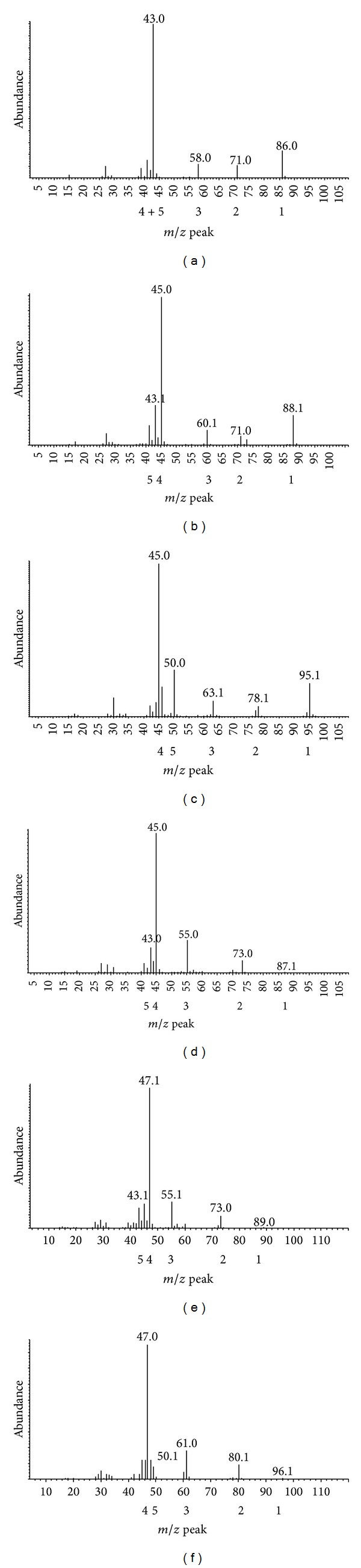
Ion fragments of the 2-pentanone peaks (a). (b), (c) and 2-pentanol peaks (d), (e), (f) peaks shown in Figure 3; (a) and (d) from ^1^H-hexanoic acid; (b) and (e) from hexanoic-2, 2-D_2_ acid; (c) and (f) from hexanoic-D_11_ acid.

Figure 4 shows the ion fragments obtained by EI MS for the unlabelled and deuterated 2-pentanone and 2-pentanol products. Five characteristic fragments were identified. Their composition is shown in Table 1.

The deuterated products were not those predicted. Those from hexanoic-D_11_ acid had nine deuterium atoms and those from hexanoic-2, 2-D_2_ acid had two. In all products, the C1 methyl group was CD_2_H.

Yagi et al. [23] challenged the assumption that methylketones are produced by *β*-oxidation and speculated that there was a different biosynthetic route. Our unexpected findings lent credence to this proposition.

### 3.4. Incubations in ^18^O_2_/^14^N_2_

Conversion of hexanoic acid to 2-pentanone by filamentous fungi is associated with a large uptake of oxygen [7]. An alternative to the proposed *β*-oxidation scheme is direct addition of molecular oxygen to hexanoic acid. We looked for incorporation of molecular oxygen in four experiments.* Penicillium roqueforti* grown on margarine was incubated for 19–23 h without (basal) and with 800 nmol of ^1^H-hexanoic acid or deuterated hexanoic acid in paired sealed vials, one of the pair in air and the other in a mixture of ^18^O_2_/^14^N_2_ (1 : 3). Incubation with hexanoic-2, 2-D_2_ acid increased 2-pentanone production by 24%, 32%, and 66% (*n* = 3) and with hexanoic-D_11_ acid by 23% to 69% (median 50%, *n* = 5). ^18^O_2_ (*m*/*z* = 36) was detectable in the HS vials at the end of the SPME analysis, confirming that ^18^O_2_ had been introduced into, and contained within, the HS incubation vials. In two experiments we set up, in parallel, positive controls in which 7,650 nmol of hexane (200 *μ*L of 38.25 mmol/L hexane in 250 mmol/L phosphate buffer pH 7.4) was incubated with a rat liver S9 preparation and NADPH-generating system for cytochrome P-450 oxygenation, using a procedure previously validated in our laboratory [24]. These vials were also incubated in air or in an ^18^O_2_/^14^N_2_ gas mixture. In both control experiments, ^18^O was incorporated into the reaction products, 2-hexanol and 2-hexanone.

The molecular mass of 2-pentanone produced from ^1^H-hexanoic acid containing ^16^O and ^18^O would be 86 and 88, respectively, from hexanoic-2, 2-D_2_ acid 88 and 90 and from hexanoic-D_11_ acid 95 and 97. For 2-pentanone produced from ^1^H-hexanoic acid, we compared the ratio of the peak areas with *m*/*z* = 88 to *m*/*z* = 86 in the vials which were incubated in ^18^O_2_, with the ratio for paired vials incubated in ^16^O_2_ to look for enrichment of the 88 ion. Similarly, we looked for evidence of enrichment of the 97 ion in vials incubated with hexanoic-D_11_ acid under ^18^O_2_. We could not use the data for hexanoic-2, 2-D_2_ acid because ^16^O D-2-pentanone has the same molecular mass (88) as ^18^O ^1^H-2-pentanone. For seven pairs of incubates with ^1^H-hexanoic acid, there was enrichment of the molecular ion for ^18^O 2-pentanone in every pair. This was extremely small (Table 2) but nonetheless was statistically significant (*P* = 0.003, *n* = 7; paired *t*-test). There was also a very small enrichment of the ^18^O molecular ion (*m*/*z* = 97) of D-2-pentanone in three incubates with hexanoic-D_11_ acid.

Because of the very low incorporation of ^18^O into 2-pentanone which was produced in large amounts in all of the above experiments, it was highly unlikely that the methylketone was formed by the direct addition of molecular oxygen to hexanoic acid or by a mixed function oxidase reaction. We cannot exclude oxygen addition by peroxidation. However, there are no double bonds in hexanoic acid, and lipase catalysed peroxidation of fatty acids adds oxygen to C1 [25] and cannot be the mechanism here.

### 3.5. Proposed Scheme for Producing 2-Pentanone-D_2_ and 2-Pentanone-D_9_ from Hexanoic-D_11_ Acid and Hexanoic-2, 2-D_2_ Acid via *β*-Oxidation

**Figure 5:**
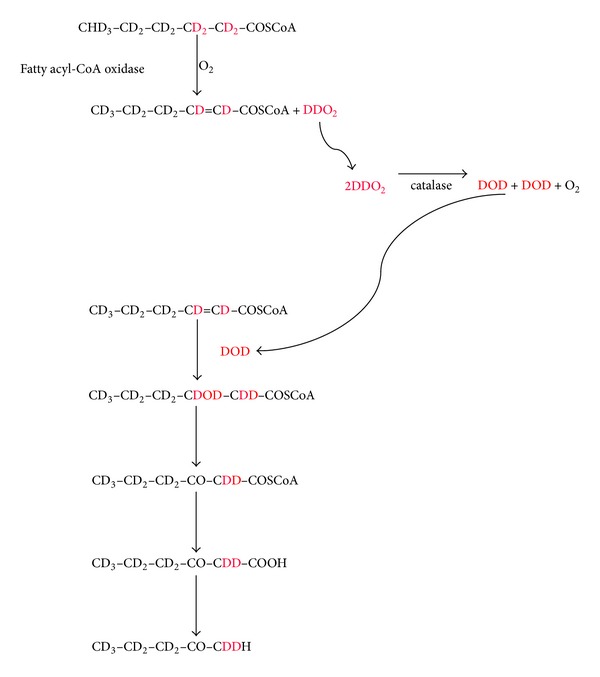
Proposed pathway for the production of 2-pentanone-D_9_ from hexanoic-D_11_ acid.

In view of all the other evidence in favour of methylketone production by *β*-oxidation, we reviewed the pathway to look for any route which would produce the deuterated products which we found. The only possibility would be if the deuterium ions transferred from carbons 2 and 3 of hexanoyl-CoA to the flavin group of acyl-CoA oxidase in the first step of the cycle were added back to the carbon skeleton later in the pathway. This could only occur when C2=C3 in 2-hexenoyl-CoA is hydrated to L-3-hydroxyhexanoyl CoA by 2-enoyl-CoA hydratase (crotonase; EC 4.2.1.17). It has now been established that the three atoms from a single water molecule are added across the double bond of 2-enoyl-CoA by 2-enoyl-CoA hydratase to form the hydrated product [14, 15]. Oxidation of hexanoyl-CoA with deuterium at C2 and C3 would yield D_2_O_2_. This, in turn, would be converted to D_2_O by catalase, which as we have shown is produced abundantly by the fungus grown on margarine. Incorporation of D_2_O into enoyl-hexanoyl-CoA would yield the observed deuterated products (Figure 5).

If correct, it would require that catalase is located very closely to acyl-CoA oxidase. This situation might have evolved to ensure immediate dismutation of H_2_O_2_ and hence protection against peroxidative damage. An apparent flaw in the argument is the low incorporation of ^18^O into 2-pentanone during incubation with ^18^O_2_/^14^N_2_. It might be predicted that at least some hydrogen removed during oxidation of hexanoyl-CoA would form H_2_^18^O_2_. For oxidation of hexanoyl-CoA, oxygen must be in direct contact with the N_5_-C_4_ flavin atoms of acyl-CoA oxidase for electron transfer [26]. It may be that ^18^O_2_ had poor access to this enzyme site because ^18^O_2_/^14^N_2_ did not displace air trapped within the fungal incubates due to inadequate mixing. Alternatively, residual ^16^O_2_ in the incubation vials, being lighter than ^18^O_2_, may have been enriched in the vapour phase and hence had better access to the enzyme [27]. A third possibility is that because bonds with lighter isotopes are broken more readily than those with heavy isotopes, ^16^O was more reactive than ^18^O in FADH_2_ oxidation [27]. If correct, our observations support other evidence that methylketone production by* Penicillium roqueforti, *other filamentous fungi, and yeasts occurs via peroxisomal *β*-oxidation [11, 13, 28]. In these organisms, production of all the *β*-oxidation enzymes increases dramatically when grown on oleate or other fatty acids as the sole carbon source. It is suppressed by growth on glucose. This response is orchestrated by fungal transcription factors, identified as Pip2p and Oaflp in the yeast* Saccharomyces cerevisiae *and farA and farB in* Aspergillus nidulans *[28]. Free CoASH released from fatty acyl-CoA intermediates by the methylketone cycle could be used by acyl-CoA oxidase and the thiolases, enabling fatty acid oxidation to continue [8]. Free CoASH diffuses poorly from the cytoplasm into peroxisomes and recycling is important [29, 30].

### 3.6. Relevance to 2-Pentanone Production in Humans

Is fatty acid *β*-oxidation the source of 2-pentanone in humans? Clinically it seems likely; firstly, excretion is increased in fasting ketosis when large amounts of fatty acids released from adipose tissue are delivered to the liver; secondly, fatty acid oxidation is induced by fasting; thirdly, this adaptive response is mediated by the transcription factor peroxisome proliferator-activated receptor alpha (PPAR-*α*), which is induced by glucocorticoids [31] and activated by fatty acids and fatty acyl-CoAs [28, 32]; fourthly, activation of PPAR-*α* increases the expression of all the enzymes of the mitochondrial and peroxisomal *β*-oxidation pathways [28, 31, 32]; and fifthly in our reported patients [1] 2-pentanone decreased rapidly in response to glucose administration. In animals, hepatic fatty acid oxidation is suppressed when fed freely [31]. 2-Pentanone is produced sometimes by the gut microflora [33, and personal observations] and this must be considered as an alternative source. However, we know nothing about the intestinal absorption of this compound, and an intestinal source would not explain the association of increased excretion with fasting ketosis or its rapid decline with glucose administration.

In humans, 2-pentanone might derive from either peroxisomal or mitochondrial *β*-oxidation. In favour of peroxisomes are firstly that peroxisomal *β*-oxidation does not proceed to completion, but stops after oxidation of C6 fatty acids [34, 35]. The specific increase in C6-derived 2-pentanone might reflect this restriction. Secondly, fasting ketosis is frequently accompanied by excretion of medium-chain dicarboxylic acids from fatty acid catabolism, which are only produced by peroxisomal *β*-oxidation [36]. The greatest increase is in hexanedioic acid. Against peroxisomal *β*-oxidation is that 2-pentanone production requires oxidation of hexanoyl-CoA, which is right at the low-end of specificity for of the mammalian straight-chain peroxisomal acyl-CoA oxidase (ACOX1). Km values of ACOX1 appear to be inversely related to the chain length of the substrates [34] and activity towards hexanoyl-CoA is very low [34, 35]. In addition, peroxisomal *β*-oxidation does not have a significant role in energy production [30].

## 4. Conclusions 

Collectively, our findings are supportive of the widely accepted view that 2-pentanone and other methylketones are produced by* Penicillium roqueforti *through incomplete metabolism of medium-chain fatty acids by *β*-oxidation, probably in peroxisomes. Our preliminary experiments corroborated the work of others. To our knowledge, our studies with deuterated hexanoic acid and ^18^O_2_ are the first to be reported. In order to explain our unexpected findings, we propose that hydrogen ions removed from hexanoyl-CoA by acyl-CoA oxidase in the first step of the pathway are cycled through hydrogen peroxide and reenter the pathway through hydration of hexenoyl-CoA. If correct, this process would protect other proteins from oxidative damage and may be relevant to the ontogeny of peroxisomes. It would also be further evidence that production of methylketones by the fungus utilises acyl-CoA oxidase and hence is peroxisomal. It is probable that 2-pentanone in humans is also produced via fatty acid catabolism. Formation through an aborted *β*-oxidation cycle similar to that used by* Penicillium roqueforti *would be consistent with the clinical observations and would contribute to CoASH recycling. Although we would favour a peroxisomal system, it could equally be a mitochondrial process in humans. A large increase in 2-pentanone in urine from an acutely sick child might be a pointer to low activity of medium-chain acyl-CoA thiolase warranting further investigation.

## Figures and Tables

**Table 1:** Fragments of 2-pentanone and 2-pentanol derived from ^1^H-hexanoic acid, hexanoic-2, 2-D_2_ acid, and hexanoic-D_11_ acid shown in Figure 4.

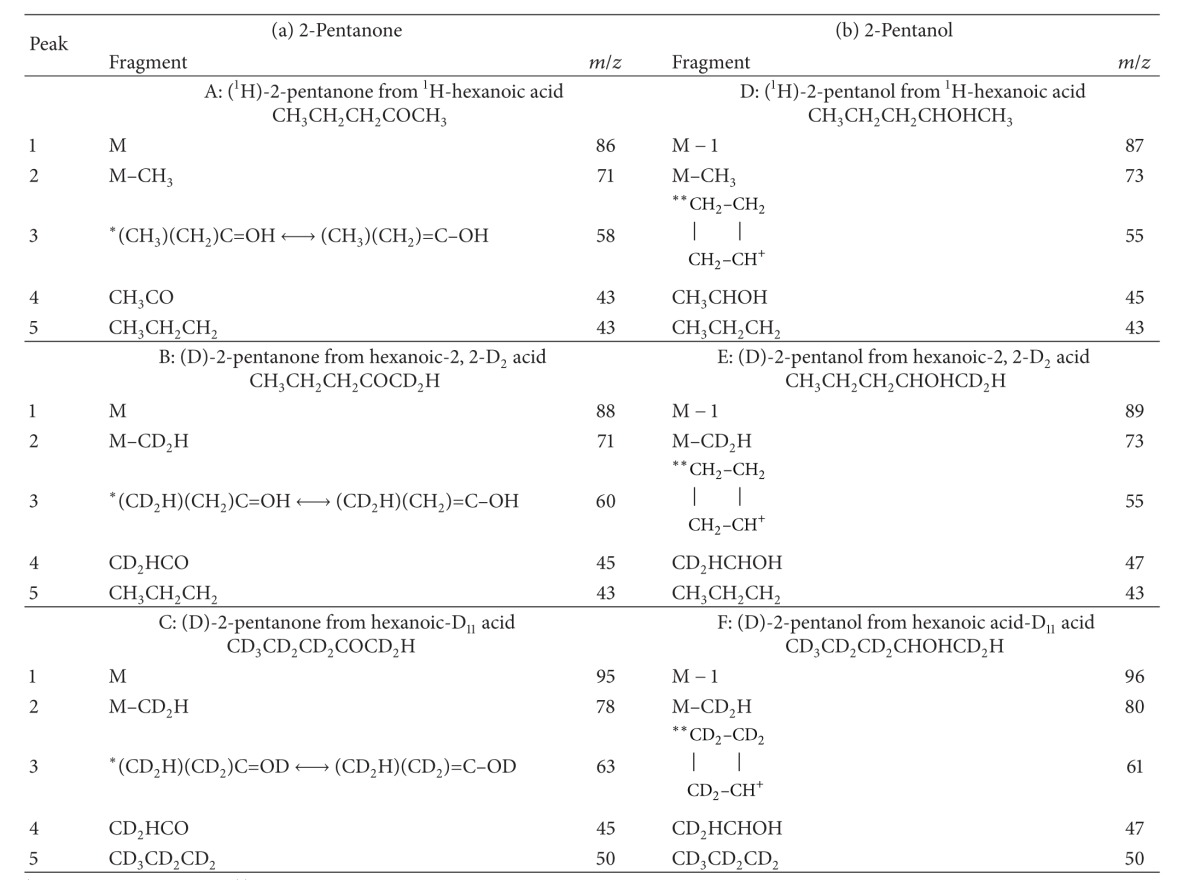

*McLafferty rearrangement. **Rearrangement.

**Table:** (a)

Paired vials	Ratio of 88/86 ions in ^18^O_2_/^14^N_2_ incubates	Ratio of 88/86 ions in air incubates
1	0.0066	0.0035
2	0.0066	0.0021
3	0.0076	0.0017
4	0.0041	0.0031
5	0.0063	0.0029
6	0.0100	0.0013
7	0.0092	0.0015

*P* = 0.0031; paired *t*-test.

**Table:** (b)

Paired vials	Ratio of 97/95 ions in ^18^O_2_/^14^N_2_ incubates	Ratio of 97/95 ions in air incubates
1	0.0069	0.0034
2	0.0043	97 ions not detected
3	0.0050	97 ions not detected
